# Clinical characteristics and radiological domains among patients with recurrent strokes-a descriptive cross-sectional study from a tertiary care center in central Nepal

**DOI:** 10.12688/f1000research.54981.1

**Published:** 2021-08-05

**Authors:** Binod Bhattarai, Shashi Bhushan Sah

**Affiliations:** 1 Neurosurgery, College of Medical Sciences, Chitwan, 0977, Nepal

**Keywords:** Recurrent, stroke, patterns, outcome

## Abstract

Background: Stroke is a significant global health hazard that ripples continuum multi-spectral effects to the patients as well their caretakers.

Methods: We studied 28 consecutive cohorts of patients with recurrent strokes managed in our centre within the last two years.

Results: The most common recurrence stroke pattern was of that of hemorrhagic to hemorrhagic subtype observed in 50% of the patients. The most common anatomical region of involvement was cortical – cortical seen in 39.28% of our cohorts. The surgical intervention was required in 17.85% whereas 42.85% of them were managed conservatively. Paradoxically, 39.28% of patients left against medical advice. The receiver operating curve (ROC) predicting mode of management was highest (area under the curve (AUC) =0.635) for compliance to therapy followed by stroke territory (AUC=0.578), age (AUC=0.457) and motor grading (AUC=0.374). The receiver operating curve (ROC) for influencing decision to leave against medical advice was highest (area under the curve (AUC) =0.861) for motor score followed by sex (AUC=0.701) and age (AUC=0.564). The analysis of variance (ANOVA) study pertaining to the mode of management was significantly connoted by the motor score and the stroke territory only. The ANOVA study pertaining to the decision to leave against medical advice was significantly governed by the motor score, stroke territory, and sex respectively. The multivariate analysis for variables governing mode of management was significant for motor score and the stroke territory only. The multivariate analysis for variables governing leave against medical advice was significant for sex, motor score and the stroke territory.

Conclusions: This study aims to appraise early dichotomization of high-risk patients for recurrent strokes to reduce the continuum of neurological events as well as to mitigate the financial aspects governing stroke care.

## Introduction

Stroke is a significant global health hazard ^1, 2^. It is a silent epidemic that ripples continuum multi-spectral effects to the patients as well their caretakers. Recurrent stroke is slowly coming out of its shell with the reported incidence of up to 22.5% within 5 years ^3^. Patients with recurrent stroke have greater disability and poorer outcomes than those with the first stroke ^4^. Amidst the current paradox between the monumental stoke economy and the dismal provisions for rehabilitative strategies; these cohorts are most often compelled to lead a poor quality of life becoming socially aloof.

The sound knowledge on the incidence and the patterns of recurrent stroke paves the way to format necessary steps in mitigating them as well as improvise newer reforms in combating them in the future. This study aims to foster the pertinent need for national stroke database studies pertaining to strokes.

## Methods

Consecutive cohorts of patients with recurrent strokes managed in the Department of Neurosurgery in College of Medical Sciences, Chitwan, Nepal within the last two years (January 2019–January 2021) were enrolled in this descriptive cross-sectional study. The recurrent stroke was defined either as:

• The stroke event occurring at the same anatomical location after 21 days of the index event or
• Different stroke event at another anatomical region within 21 days of the index event ^5^.

Current smokers were defined as those who have smoked ≥1 cigarette per day for 6 months and have smoked in the last 28 days, whereas heavy drinkers were labeled as those who have consumed >2 and >1 standard drink per day (a glass of wine, a bottle of beer, or a shot of spirits, ∼10 to 12 g of ethanol) for men or and women respectively ^6^. Measurement of treatment adherence (compliance) was performed by the self reported and pills count methods.

The anatomical regions of involvement with the recurrent events were further categorized into subgroups as:

• Cortical and cortical
• Cortical and basal ganglionic
• Cortical and thalamic
• Basal ganglion and basal ganglionic
• Basal ganglionic and thalamic and
• Thalamic and thalamic

The demographical and the clinico-radiological variables comprising of age, sex, presenting clinical motor score, medical compliance, patterns of the index and recurrent strokes, anatomical distribution of the strokes, the mode of management, and those who left against medical advice (LAMA) were thoroughly appraised and analyzed.

The ‘equipoise’ governed from the recurrent events occurring in the same patient within the same geographical minimized the bias on the outcome from other confounding factors such as alcohol intake, smoking status and other medical comorbidities in our cohort study.

### Inclusion criteria

• Consecutive 28 patients presenting with recurrent strokes in our Neurosurgical unit

### Exclusion criteria

• Failure of approval for participation in the study
• Simultaneous multiple intracerebral strokes
• Traumatic ICH
• ICH secondary to vascular malformation, aneurysm, or cavernoma.
• Transient ischemic attacks (TIA)
• Patients with missing data pertaining to the study variables

The sample size required for adequate statistical elaboration was calculated according to the formula

n = Z2 × p × q/d2 where

Z=1.96 at 95% confidence interval,

p =7.4% prevalence of recurrent CVA ^5^

q =1–p and

d=10% margin of error

The required sample size calculated was 26.32

We analyzed the records of 28 patients.

Frequency distribution (counts and percentages) was undertaken for the studied variables of the cohorts included in our study. Data were recruited and analyzed using the SPSS version 16 software. Statistical analysis was done utilizing receiver operating curve (ROC) with area under curve (AUC) values, Analysis of variance (ANOVA) and multivariate logistic regression analysis among the pertinent variables applying mode of management and the decision to leave against medical advice as the final outcomes. P-value of <0.05 was considered significant. Patients who had missing data for variables were excluded from the analysis.

### Ethical approval

All procedures performed in studies involving human participants followed the ethical standards of the institutional and/or national research committee and with the 1964 Helsinki Declaration and its later amendments or comparable ethical standards. The study was approved by the Institutional Review Committee (IRC) of the College of Medical Sciences (COMS-IRC-2021-35), Chitwan in Nepal.

### Consent

The article was based on a review of anonymous clinical data. Therefore, no patient consent was required.

## Results

In our study, the prevalence of recurrent stroke was more common among male cohorts (M: F ratio of 1.54:1). The mean age of presentation of the studied population was 67.65 ±10.39 for males and 61.64 ±13.54 for females respectively.

Medical compliance was observed in only 64.28% of the patients. The smoking and alcohol consumption habit was seen in 35.71% and 64.28% of patients respectively.

The mean motor score of the patient at presentation was 1.46 ±0.63.

The recurrence pattern was observed to be of hemorrhagic to hemorrhagic (H-H) in 14/28 (50%), ischemic to hemorrhagic (I-H) in 12/28 (42.85%), ischemic to ischemic (I-I) in 2/28(7.14%) of the cohorts.

The pattern of recurrent stroke pertaining to the anatomical distribution was cortical – cortical in 11/28 (39.28%), cortical –basal ganglia in 2/28 (7.14%), basal ganglion- basal ganglion in 9/28(32.14%), basal ganglion-thalamic in 3/28(10.71%), thalamic–thalamic in 2/28 (7.14%) and cortical-thalamic in 1/28 (3.57%) of our cohort study.

The surgical intervention was required in 5/28 (17.85%) whereas 12/28(42.85%) of them were managed conservatively. Paradoxically, 11/28(39.28%) of patients left against medical advice. The relevant findings of our study have been summarized in Table 1.

**Table 1.** Summary of pertinent findings in our study.

Variables	Frequency
Male: Female	1.54:1
Mean Age (years)	Male-67.65 ±10.39 Female-61.64 ±13.54
Medical Compliance	18 (64.28%)
Smoking Status	10(35.71%)
Alcohol Intake	18(64.28%)
Mean Motor Score at Presentation	1.46 ±0.63
**Patterns of presentation**	
Hemorrhagic-Hemorrhagic	14(50%)
Ischemic-Hemorrhagic	12 (42.85%)
Ischemic -Ischemic	2 (7.14%)
**Anatomical Categorization**	
Cortical-Cortical	11 ±0.67 (39.28%)
Cortical –Basal Ganglion	2 ±0 (7.14%)
Basal Ganglion-Basal Ganglion	9 ±0.72 (32.14%)
Basal Ganglion-Thalamic	3 ±0.57 (10.71%)
Thalamic-Thalamic	2 ±0.63 (7.14%)
Cortical-Thalamic	1 ±0 (3.57%)
**Management (Index Event)**	
Surgical	5 (17.85%)
Medical	12 (42.85%)
Leave Against Medical Advice	11 (39.28%)

**Figure 1.**
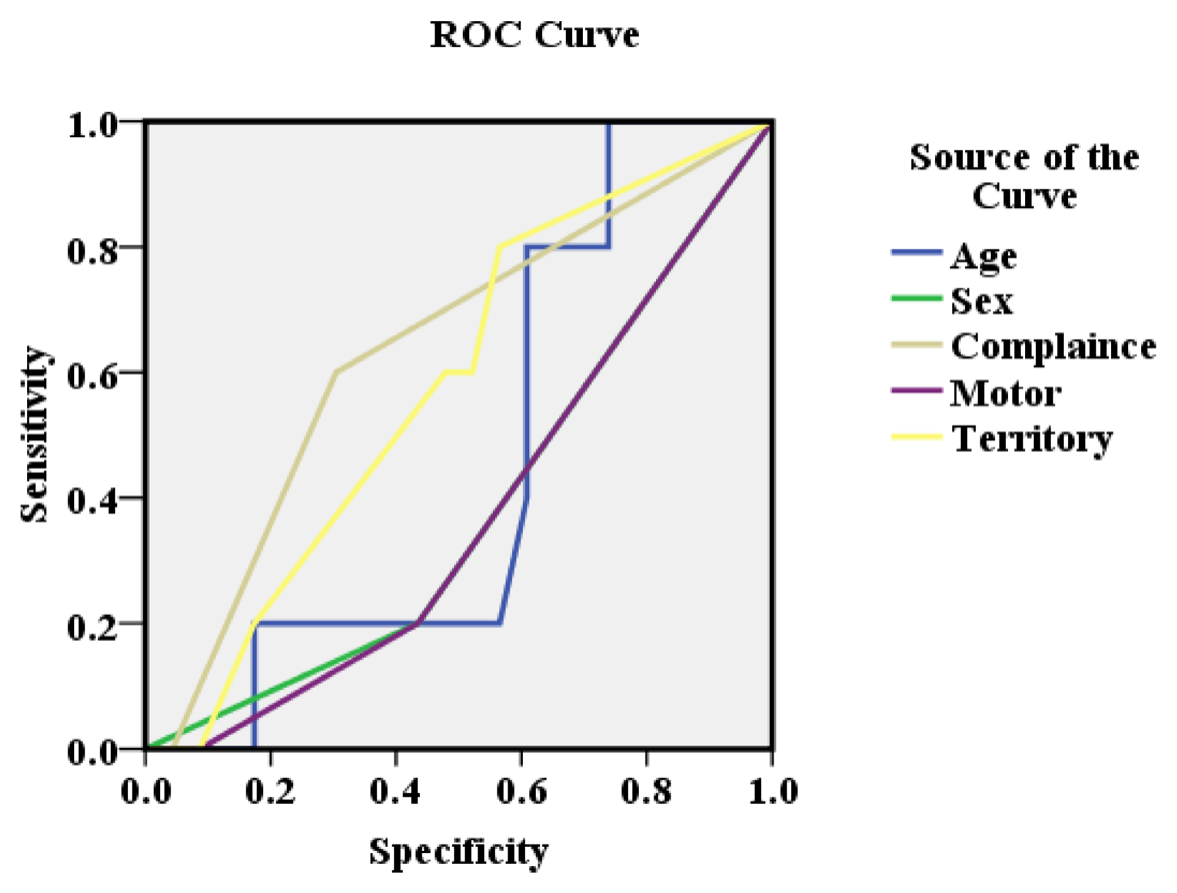
The receiver operating curve (ROC) of the variables governing mode of management.

The receiver operating curve (ROC) of the study for predicting mode of management was highest (area under the curve (AUC) =0.635) for compliance to therapy followed by stroke territory (AUC=0.578), age (AUC=0.457) and motor grading (AUC=0.374) as shown in Figure 1.

**Figure 2.**
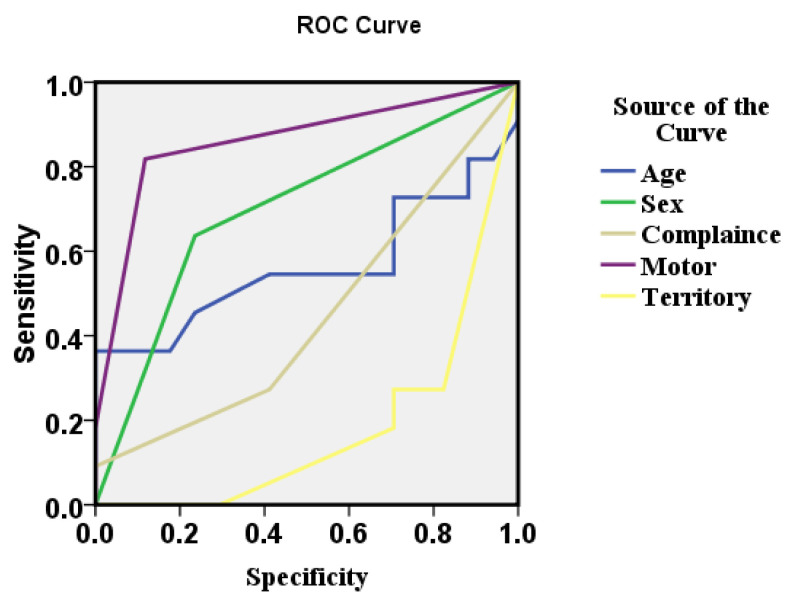
The receiver operating curve (ROC) of the variables governing leave against medical advice.

The receiver operating curve (ROC) of the study for influencing decision to leave against medical advice was highest (area under the curve (AUC) =0.861) for motor score followed by sex (AUC=0.701) and age (AUC=0.564) as shown in Figure 2.

The ANOVA study pertaining to the mode of management was significantly connoted by the motor score and the stroke territory only as shown in Table 2.

**Table 2.** ANOVA analysis of variables governing mode of management.

		Sum of Squares	df	Mean Square	F	Sig.
Motor	Between Groups	5.200	1	5.200	23.451	.000
	Within Groups	5.765	26	.222		
	Total	10.964	27			
Territory	Between Groups	24.614	1	24.614	10.655	.003
	Within Groups	60.064	26	2.310		
	Total	84.679	27			
Ictus event	Between Groups	.037	1	.037	.140	.712
	Within Groups	6.963	26	.268		
	Total	7.000	27			

The ANOVA study pertaining to the decision to leave against medical advice was governed by the motor score, stroke territory, and sex as shown in Table 3. 

**Table 3.** ANOVA analysis of variables governing leave against medical advice.

		Sum of Squares	df	Mean Square	F	Sig.
Motor	Between Groups	5.200	1	5.200	23.451	.000
	Within Groups	5.765	26	.222		
	Total	10.964	27			
Territory	Between Groups	24.614	1	24.614	10.655	.003
	Within Groups	60.064	26	2.310		
	Total	84.679	27			
Ictus event	Between Groups	.037	1	.037	.140	.712
	Within Groups	6.963	26	.268		
	Total	7.000	27			
Age	Between Groups	2.228	1	2.228	.015	.903
	Within Groups	3803.487	26	146.288		
	Total	3805.714	27			
Sex	Between Groups	1.074	1	1.074	4.984	.034
	Within Groups	5.604	26	.216		
	Total	6.679	27			

The multivariate analysis for variables governing mode of management was significant for motor score and the stroke territory as shown in Table 4.

**Table 4.** Multivariate analysis of variables governing mode of management.

Source	Dependent Variable	Type III Sum of Squares	df	Mean Square	F	Sig.
Corrected Model	Age	8.502 ^a^	2	4.251	.028	.972
	Sex	1.083 ^b^	2	.542	2.420	.109
	Motor	5.248 ^c^	2	2.624	11.474	.000
	Territory	26.030 ^d^	2	13.015	5.548	.010
Management	Age	8.502	2	4.251	.028	.972
	Sex	1.083	2	.542	2.420	.109
	Motor	5.248	2	2.624	11.474	.000
	Territory	26.030	2	13.015	5.548	.010

The multivariate analysis for variables governing leave against medical advice was significant for sex, motor score and the stroke territory as shown in Table 5.

**Table 5.** Multivariate analysis of variables governing leave against medical advice.

Source	Dependent Variable	Type III Sum of Squares	df	Mean Square	F	Sig.
Corrected Model	Age	2.228 ^a^	1	2.228	.015	.903
	Sex	1.074 ^b^	1	1.074	4.984	.034
	Motor	5.200 ^c^	1	5.200	23.451	.000
	Territory	24.614 ^d^	1	24.614	10.655	.003
LAMA	Age	2.228	1	2.228	.015	.903
	Sex	1.074	1	1.074	4.984	.034
	Motor	5.200	1	5.200	23.451	.000
	Territory	24.614	1	24.614	10.655	.003

## Discussion

Stroke is a significant global health hazard ^7^. The continuum impact of the associated morbidity and mortality is mostly observed in low and middle-income nations ^6^. With the concurrent increment in the lifespan of the population alongside adaptations of unhealthy lifestyle and living habits, the prevalence of stroke will certainly show an upward curve in the coming future ^7^.

The reported incidence of recurrent stroke despite preventive measures is above 20% ^8^. The risk of such adverse events is estimated at 5.4% and 11.3% during the first and the fifth years respectively, with an overall risk of 1.2/100/year for both hemorrhagic and the ischemic subtypes ^9, 10^.

The ‘‘cortical–cortical’’ is the most common pattern of anatomical involvement in recurrent hemorrhagic strokes. This has largely attributed secondary to cerebral amyloid angiopathy and coagulation disorders ^10^. The similar anatomical pattern of involvement (39.28%) was seen in our study. The hemorrhagic-hemorrhagic patterns of the index and the recurrent stroke events were observed in 50% of the cohorts.

Hypertension is the strongest risk factor for both subtypes of recurrent stroke with an overall relative risk of almost 5.43 ^7, 9^. Similarly concurrent transient ischemic events and the radiological presence of chronic infarction have shown to prognosticate increased odds of recurrent ischemic strokes ^5^. Such epiphenomenon have been shown to increase the mortality risk by almost 17 folds ^9^. Increased age is also a risk variable for stroke recurrence with the estimated hazard ratio of 1.02/year ^9^. The average age of patients in a study ranged from 54 to 66 years ^10^. However, both age and sex were not found to be linked to harbinger subsequent IS ^10^. Male genders have shown to have a higher cumulative risk for recurrent stroke ^11^. Our study showed male gender preponderance with a ratio of 1.54:1. Age ≥65 years have been found to be an independent predictor of long-term mortality ^12^. The average age of the patients in our cohort study was 65.45 years. In addition to having higher stroke risk, women have poorer post-stroke outcomes. However, in the literature, these differences in sex are not consistent ^12^.

Smoking habit has been attributed as a risk factor for ischaemic strokes only ^7^. The smoking status was observed in 35.71% whereas the alcohol intake habit was seen in 68.28% of patients. Hypertension and diabetes mellitus are other documented independent risk factors. Moreover, hypertension at admission during index stroke events prognosticate the risk of early mortality ^12^.

One study showed that only 45% of patients were aware of their hypertension, and approximately 30% of them were compliant with their medications ^7^. Another study revealed that only 12% of patients with atrial fibrillation were receiving appropriate prophylactic anticoagulation therapy ^9^. In our study, adherence to medication was found in 64.28% of patients.

The neurological improvement to compensate for carrying out activities of daily living is further compromised among patients with contralateral recurrence, further hampering their quality of life ^8^.

Mortality among patients with recurrent strokes have been documented to be above 35% with a hazard ratio of 2.55 ^9^. The all-cause mortality at five years pertaining to hemorrhagic strokes is almost twice comparing to the ischemic counterpart. The result mirrors the risk of mortality among patients with non-lacunar infarction comparing to that of the lacunar subtypes ^11^.

Patients with large vessel atherosclerosis and cardio-embolism have an early risk of stroke recurrence ^13^. 

The yearly estimates of recurrent stroke, death, and cardiovascular events were reported at 3.6%, 10.5%, and 6.7%, respectively in one study ^14^. The 5-year rate event for MI is 41% for recurrent stroke comparing to only 2% after the first stroke. The cumulative event rates for major vascular events are 18% and 45% at 1 and 5 years respectively ^11^.

The pattern of stroke recurrence mirrored that of the index events more for the ischemic subtypes, compared to their hemorrhagic counterparts (90% Vs. 56%) ^11^.

Patients managed in dedicated stroke units have shown to have improved outcome ^5^. However; the expenditure pertaining to stroke care has been projected to cross 150 billion dollars by 2030 ^15^. One of the salient findings in our study was the observation of almost 40% of patients who opted to leave against medical advice from the hospital. This reflects the poor perception about the disease and its impact upon the patient by their caretakers’ superadded by the financial burden associated with stroke care. This further reinforces the imminent need of implementing primary and secondary preventive measures to prevent ad mitigate such events. 

Plaque stability in the extracranial group, whereas the progression of stenosis in the intracranial group determines the risk of recurrence ^16^. Among stroke with no determined cause, intracranial stenosis was often found at the time of recurrence ^16^. Timely and appropriate screening of these high-risk patients helps to reduce the short and long-term multispectral neurological and financial burden among such patients as well as their caretakers.

## Limitations of the study

The true estimates of the recurrence may be underestimated owing to omission bias since our study reviewed medical records of admitted patients within the last two years only. We also have scarce data pertaining to the analysis of factors governing long-term morbidity and mortality. Finally, the results of our study may not mirror demographics from other topography. 

## Conclusion

A provision for nationwide hospital and community-based stroke register is therefore of paramount importance to monitor the patterns and quality of primary and recurrent stroke care. The early dichotomization of high-risk patients for recurrent strokes is essential to reduce the continuum of neurological events as well as to mitigate the financial aspects governing stroke care. This is even more relevant in our context wherein there is a high stroke burden with paradoxical minimal stroke care and rehabilitative facilities.

## Data availability

Figshare. Clinical characteristics and radiological domains among patients with recurrent strokes-a descriptive cross-sectional study from a tertiary care center in central Nepal ^17^. DOI: https://doi.org/10.6084/m9.figshare.14923071.v1

Data are available under the terms of the Creative Commons Zero "No rights reserved" data waiver (CC BY 4.0 Public domain dedication).
