# Supplementary material for: Clinical characteristics and radiological domains among patients with recurrent strokes-a descriptive cross-sectional study from a tertiary care center in central Nepal
Source: F1000Res. 2021 Aug 5;10:757. [Version 1] doi: 10.12688/f1000research.54981.1 (PMC8581597; doi:10.12688/f1000research.54981.1)
Supplement: Peer review history

# Peer review: Reviewer response for version 1

**DOI:** 10.5256/f1000research.58514.r91259

**Published:** 2021-11-10

**Authors:** Guoyi Gao

**Affiliations:** 1 Department of Neurosurgery, Shanghai Jiao Tong University, Shanghai, China

This is an interesting report on recurrent stroke in one center in Nepal.  I believe that many colleagues are keen to learn the current status of neurological diseases and the treatment in this Southern Asia area. Thus I suggest this manuscript is suitable for indexing after revision.

1. The inclusion criteria could be rewritten.
2. In the demographic variables, I did not find the preexisting condition of hypertension, as well as the antihypertensive treatment. The author could add this information to strengthen the evidence. 
3. The author should not neglect that the major limitation of this study is the small sample, this could be described in the part of limitations. 

Is the work clearly and accurately presented and does it cite the current literature?

Yes

If applicable, is the statistical analysis and its interpretation appropriate?

Yes

Are all the source data underlying the results available to ensure full reproducibility?

Yes

Is the study design appropriate and is the work technically sound?

Yes

Are the conclusions drawn adequately supported by the results?

Yes

Are sufficient details of methods and analysis provided to allow replication by others?

Yes

Reviewer Expertise:

Neurosurgery

I confirm that I have read this submission and believe that I have an appropriate level of expertise to confirm that it is of an acceptable scientific standard.

# Peer review: Reviewer response for version 1

**DOI:** 10.5256/f1000research.58514.r92803

**Published:** 2021-08-31

**Authors:** Lekhjung Thapa

**Affiliations:** 1 Department of Neurology, National Neuro Center (NNC), Maharajgunj, Kathmandu, Nepal

This is a good report highlighting characteristics of a group of stroke patients who presented to a neurosurgical unit of a tertiary care center with hemorrhagic stroke in 21 days, which the authors have considered as a recurrent stroke. Although the community prevalence of stroke report in Nepal is available ^1^, the prevalence of recurrent stroke is still lacking. The authors have used data from an international study to calculate the sample size and have studied 28 patients with hemorrhagic stroke who had an ischemic or hemorrhagic stroke in the past 21 days. After reading the article, I found it very interesting that a very high proportion (40%) of the patients left against medical advice which is very common in developing countries because of various unavoidable reasons like a financial burden, and moreover, a grim prognosis associated with recurrent stroke. This information highlights not only the need for a stroke registry as the authors have highlighted but also for secondary stroke prevention awareness programs. ^2^ Few suggestions for authors willing to conduct a similar study would be:

1. Incorporation of important clinical variables that are known to be risk factors for recurrent stroke such as data on hypertension, metabolic syndrome, dietary habits, stress, etc.
2. ICH score would be a more meaningful way to communicate in cases of ICH instead of motor score
3. Cardio-embolism (Rheumatic Heart Disease) is a unique cause of recurrent stroke in developing countries like Nepal and a note on it would be better as there is no risk

Is the work clearly and accurately presented and does it cite the current literature?

Yes

If applicable, is the statistical analysis and its interpretation appropriate?

I cannot comment. A qualified statistician is required.

Are all the source data underlying the results available to ensure full reproducibility?

Yes

Is the study design appropriate and is the work technically sound?

Yes

Are the conclusions drawn adequately supported by the results?

Yes

Are sufficient details of methods and analysis provided to allow replication by others?

Yes

Reviewer Expertise:

Stroke

I confirm that I have read this submission and believe that I have an appropriate level of expertise to confirm that it is of an acceptable scientific standard.

## References

[1] : Prevalence of Stroke and Stroke Risk Factors in a South-Western Community of Nepal. *J Stroke Cerebrovasc Dis* .2021;30(5) : 10.1016/j.jstrokecerebrovasdis.2021.105716 105716 10.1016/j.jstrokecerebrovasdis.2021.105716 33725500

[2] Stroke: Knowing it better makes a difference. *Forbes India* .2021; Reference source
